# A non-canonical adenosinergic pathway led by CD38 in human melanoma cells induces suppression of T cell proliferation

**DOI:** 10.18632/oncotarget.4693

**Published:** 2015-07-25

**Authors:** Fabio Morandi, Barbara Morandi, Alberto L. Horenstein, Antonella Chillemi, Valeria Quarona, Gianluca Zaccarello, Paolo Carrega, Guido Ferlazzo, Maria Cristina Mingari, Lorenzo Moretta, Vito Pistoia, Fabio Malavasi

**Affiliations:** ^1^ Laboratory of Oncology, Istituto Giannina Gaslini, Genoa, Italy; ^2^ Department of Experimental Medicine, University of Genoa, Genoa, Italy; ^3^ Department of Medical Sciences, Laboratory of Immunogenetics and CeRMS, University of Torino, and Transplant Immunology, Città della Salute e della Scienza, Torino, Italy; ^4^ Istituto Giannina Gaslini, Genoa, Italy; ^5^ Department of Human Pathology, University of Messina, Italy; ^6^ Cellular Therapy Program, University Hospital - A.O.U. Policlinico, Messina, Italy

**Keywords:** Melanoma, ectoenzymes, adenosine, immunosuppression

## Abstract

Nucleotide-metabolizing ectoenzymes are endowed with an extracellular catalytic domain, which is involved in regulating the extracellular nucleotide/nucleoside balance. The tumor microenvironment contains high levels of adenosine (ADO) generated by this enzymatic network, thus promoting tumor growth by inhibiting anti-tumor immune responses. ADO inhibition in melanoma murine models limits tumor metastases and restores anti-tumor immune responses.

This work investigates the expression and function of ectoenzymes in primary human melanoma cell lines. All of latter cells expressed CD38, CD39, CD73, and CD203a/PC-1, and produced ADO from AMP and NAD^+^. Melanoma cells inhibited T cell proliferation through an ADO-dependent mechanism, since such inhibition was reverted using CD38/CD73 specific inhibitors.

Melanoma cells abolished the function of effector memory, central memory and reduced naïve CD4^+^ T cell proliferation. Accordingly, phosphorylation of S6 ribosomal protein, p38 and Stat1 was lower in activated memory cells than in naïve CD4^+^ T lymphocytes. Melanoma cells also inhibited proliferation of naïve, memory and -to a lesser extent- of effector CD8^+^ T cells. These different inhibitory effects correlated with distinct patterns of expression of the ADO receptor A2a and A2b. These results show that primary human melanoma cell lines suppress *in vitro* T cell proliferation through an adenosinergic pathway in which CD38 and CD73 play a prominent role.

## INTRODUCTION

The adenosine (ADO) nucleoside is generated from the catabolism of several nucleotides (*i.e*. ATP, ADP, ADPR and AMP) or of NAD^+^ by nucleotide-metabolizing ectoenzymes. These ectoenzymes are expressed on the outer membrane of numerous cell populations and exert their enzymatic activity in the extracellular milieu. The canonical pathway of ADO production starts from CD39, an ecto-nucleoside triphosphate diphosphohydrolase (NTPDase), which converts ATP to ADP and then into AMP. The latter molecule is further converted to ADO by the 5′-nucleotidase (5′-NT) CD73 [1]. An alternative pathway has been recently demonstrated. The key molecule is CD203a/PC-1, since it converts both ADPR (generated from NAD^+^ by CD38) or ATP to AMP, that is subsequently metabolized into ADO by CD73 [2, 3].

Under physiological conditions, the concentration of ADO in the extracellular environment is normally very low. However, the presence of high levels of ADO in pathological conditions, such as tissue injury, ischemia, hypoxia, inflammation, trauma and cancer [4] activates the specific adenosine receptors (ADOR) on immune cells, giving rise to immunosuppressive effects [5, 6]. Specific ligation of ADORA2a inhibits NK cell cytotoxicity and IFN-γ release [4], while engagement of ADORA2a and ADORA2b dampens T lymphocyte effector functions (mainly cytotoxicity and cytokine release [4, 7, 8]. Moreover, ADO inhibits macrophage activation by interacting with ADORA2a and induces M2 polarization *via* ADORA2b. Finally, activation of ADORA2b hinders dendritic cells maturation and differentiation, leading to defective antigen presentation [4].

ADO is released in the neoplastic microenvironment either by CD73^+^ tumor cells or by CD73^+^ infiltrating leukocyte subsets, such as myeloid-derived suppressor cells (MDSC) or regulatory T cells (Treg) [4, 9, 10]. Several studies reported that elevated expression of CD73 by tumor cells correlated to a worse prognosis of patients with different types of solid tumors, such as breast cancer [11], melanoma [12, 13], prostate cancer [14] and gastric carcinoma [15]. High concentrations of ADO are present in the tumor microenvironment also in murine models [16]. In line with this, blockade or inhibition of CD73 [6, 10, 17–21], CD39 [22, 23] and ADORs [6, 11, 24–27] in the same models resulted in the reduction of tumor growth and metastasis.

Primary cell lines previously generated in our laboratory from melanoma biopsies [28] inhibit NK cell functions through the production of immunosuppressive molecules such as IDO and PGE_2_ [29].

So far, CD73 is the only component of the ectoenzymatic pathways of ADO production whose expression has been reported in human melanoma cells [12]. No information is available regarding the expression and function of the other ectoenzymes involved (CD38, CD39 and CD203a/PC-1). Several groups have demonstrated that melanoma cells can inhibit T cell function, mainly via PD-1/PDL-1 interaction [30–32]. Interestingly, PD-1 expression on malignant cells is induced by hypoxia [31], similarly to what observed for ADO. Moreover, both molecules can be expressed or released also by cells infiltrating the tumor microenvironment (i.e. Treg) [32].

This study demonstrated that i) high amounts of ADO are generated by malignant melanoma cells through both the canonical and non-canonical ectoenzymatic pathways, and ii) ADO produced by melanoma cells exerts differential influence on the T lymphocyte populations involved in the anti-tumor immune response.

## RESULTS

### Melanoma cell lines express nucleotide-metabolizing ectoenzymes

The first step in this study was to analyse the expression of a panel of ectoenzymes on six primary melanoma cell lines (MECA, METRAV, MEPA, MECO, MEMO and MEOL), using a commercially available melanoma cell line (FO1) as control.

**Figure 1:**
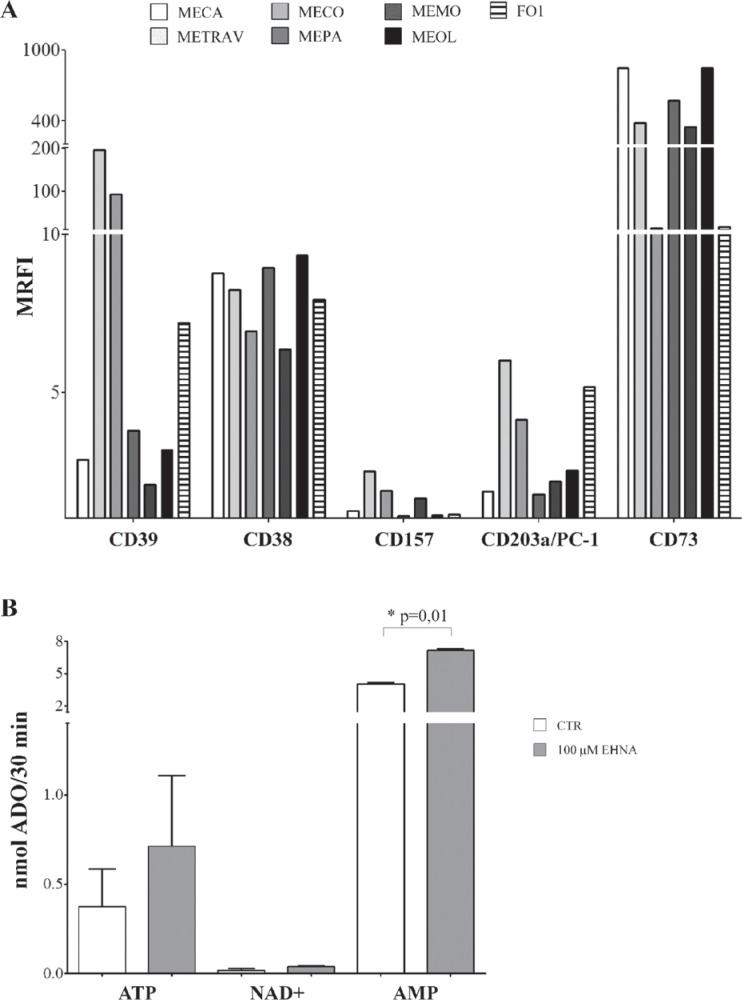
**Panel A. Ectoenzyme expression on melanoma cells lines.** The expression of CD39, CD38, CD157, CD203a/PC-1 and CD73 was assessed by flow cytometry line on the 6 primary melanoma cell lines (METRAV, MECA, MECO, MEPA, MEMO, MEOL) and on the FO1 melanoma cell line. Data are expressed as MRFI. **Panel B.** ADO production by melanoma cell lines. ADO production was investigated by HPLC in supernatants from four melanoma cell lines incubated with different substrates (ATP, NAD^+^ and AMP at 100 μM), in the presence (grey bars) or absence (white bars) of EHNA. Data are expressed as nmol ADO/30 min ± SD. *p* values are indicated where differences are statistically significant.

Figure 1, panel A, shows that CD39 was highly expressed by two primary cell lines (METRAV and MECO, MRFI 196.63 and 96.13, respectively), but only moderately expressed in the other cell lines (MRFI range 2.07–7.18). CD38 was expressed by all cell lines analyzed (MRFI range 6.36–9.35), while CD157 expression was barely detectable (MRFI range 1.07–2.48). CD203a/PC-1 was expressed by all melanoma cell lines (MRFI range 1.77–6) with a high expression on METRAV (MRFI 6), MECO (MRFI 4.14), and FO-1 cell lines. The expression of CD73, the enzyme that leads to ADO production in both pathways, was very high in all cell lines examinated (MRFI range 14.17–849.13).

These observations indicated that melanoma cells are equipped with the complete set of molecules constituting the canonical (CD39/CD73) and alternative (CD38/CD157/CD203a(PC-1)/CD73) pathways for ADO production.

### ADO production by primary melanoma cell lines

Next, we asked whether the ectoenzyme pathways expressed by melanoma cells were functional. To this end, we selected two CD39^high^/CD203a(PC-1)^high^ (METRAV, MECO) and two CD39^low^/CD203a(PC-1)^low^ (MECA and MEOL) melanoma cell lines and evaluated the production of ADO in cell culture supernatants upon addition of ATP, NAD^+^ or AMP (100 μM) as substrates. Cells were cultured in the presence or absence of EHNA, an inhibitor of ADO deaminase enzyme.

Figure 1, panel B, shows that the highest production of ADO was obtained when AMP served as the substrate (mean nmol ADO/30 min ± SD: 4.05 ± 0.28). Furthermore, ADO production was significantly increased in the presence of EHNA (7.16 ± 0.32, *p* = 0.01). These results indicated that ADO is produced and then partially consumed by ADO deaminase. In contrast, ADO production was low when using ATP (0.37 ± 0.41) or NAD^+^ (0.017 ± 0.02) as substrates. In both cases, ADO production was almost unaffected by EHNA treatment. These observations suggest that CD73 is functional on melanoma cells, since AMP is converted to ADO with high efficiency. The low levels of ADO production by melanoma cells observed after providing NAD^+^ as substrate may indicate that enzymatic function either of CD38 or CD203a/PC-1, which converts ADPR produced from NAD^+^ by CD38 to AMP, is inefficient. The latter possibility appears to be more likely, since the production of ADO from ATP (initiated by CD39) is also low. It is fair to assume that enzymatic activities of these molecules in melanoma cells are limited.

**Figure 2:**
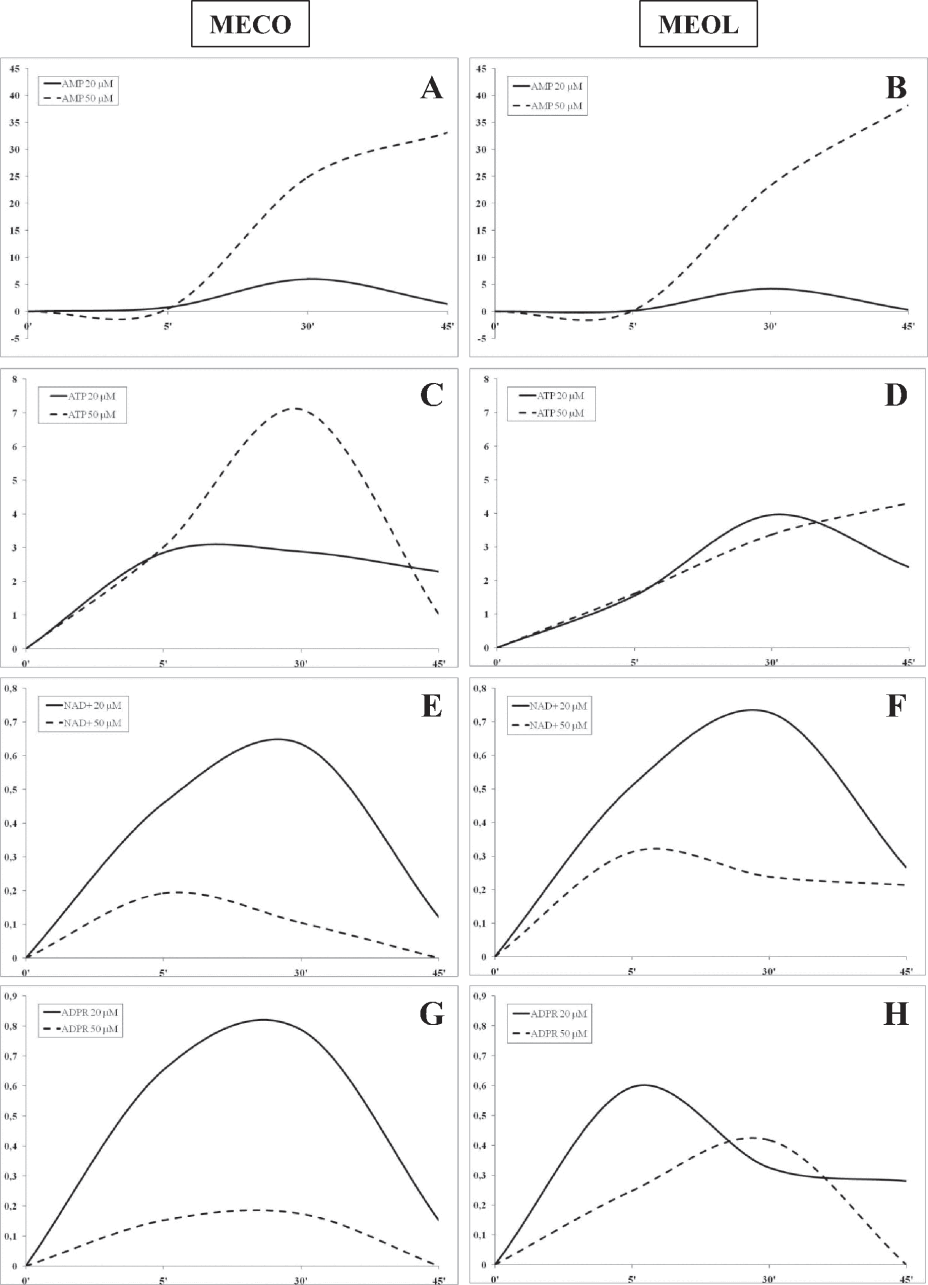
Kinetics of ADO production by melanoma cell lines ADO production was investigated in supernatants collected at 5, 30 and 45 min from MECO (**panel A-C-E-G**) and MEOL (**panel B-D-F-H**) cell lines treated with AMP (**panel A-B**), ATP (**panel C-D**), NAD^+^ (**panel E-F**) and ADPR (**panel G-H**) 20 (continuous lines) or 50 (dotted lines) μM. Data are expressed as nmol ADO.

We then turned our attention to analyse the kinetics of ADO production, using different concentrations of ATP, AMP, NAD^+^ and ADPR (20 and 50 μM). To this aim, we selected one CD39^high^CD203a(PC-1)^high^ (MECO, Figure 2, panels A, C, E and G) and one CD39^low^CD203a/PC-1^low^ (MEOL, Figure 2, panels B, D, F, and H) melanoma cell line.

The highest level of ADO production was achieved using AMP as substrate in both cell lines, with a peak at 30 min using 20 μM AMP (MECO 6 nmol ADO, MEOL 4.2 nmol ADO) and a linear increase using 50 μM AMP (MECO 33.17 nmol ADO/45 min, MEOL 38.2 nmol ADO/45 min) (Figure 2, panels A and B). ADO production from NAD^+^ or ADPR was extremely low. Both cell lines peaked at 5 min using 50 μM NAD^+^ (MECO 0.19 nmol ADO, MEOL 0.31 nmol ADO) and at 30 min using 20 μM NAD^+^ (MECO 0.63 nmol ADO, MEOL 0.72 nmol ADO). In contrast, MECO peaked at 30 min using 20 μM (0.78 nmol ADO) or 50 μM ADPR (0.17 nmol ADO), whereas MEOL displayed a peak at 5 min using 20 μM ADPR (0.59 nmol ADO) and another peak at 30 min using 50 μM ADPR (0.41 nmol ADO). For both cell lines, the highest production of ADO was achieved when using the lowest concentration of substrates, thus suggesting that their enzymatically active sites may be inactivated and/or saturated by ADPR or NAD^+^ at high concentrations.

ADO production was similar in the two melanoma cell lines when using 20 μM ATP as substrate: the peak was reached in 5 min in MECO (2.89 nmol ADO) and in 30 min in MEOL (3.96 nmol ADO) followed by a slow decline in both. In contrast, the use of 50 μM ATP led to different results: MECO displayed a peak at 30 min (7.1 nmol ADO) followed by rapid decrease, while MEOL showed a linear increase (4.32 nmol ADO/45 min). This different kinetics reflect the different expression of CD39 and CD203a(PC-1) by the two cell lines (see Figure 1, panel A).

### Melanoma cells inhibited T cell proliferation

To test the possibility that melanoma cell lines inhibited T cell proliferation through ADO production, we evaluated the proliferation of CD4^+^ and CD8^+^ T cells activated by anti-CD3/anti-CD28 beads in the presence or absence of primary melanoma cells. The effect was quantified using a CFSE dilution assay. In some experiments, melanoma cells were pre-treated with kuromanin (inhibitor of CD38), dipyridamole (inhibitor of nucleoside transporter), α-β-methyl ADP (inhibitor of CD73) and β-γ-methyl ATP (inhibitor of CD203a/PC-1), to assess the individual contribution of each ectoenzyme in inhibiting T cell proliferation.

**Figure 3:**
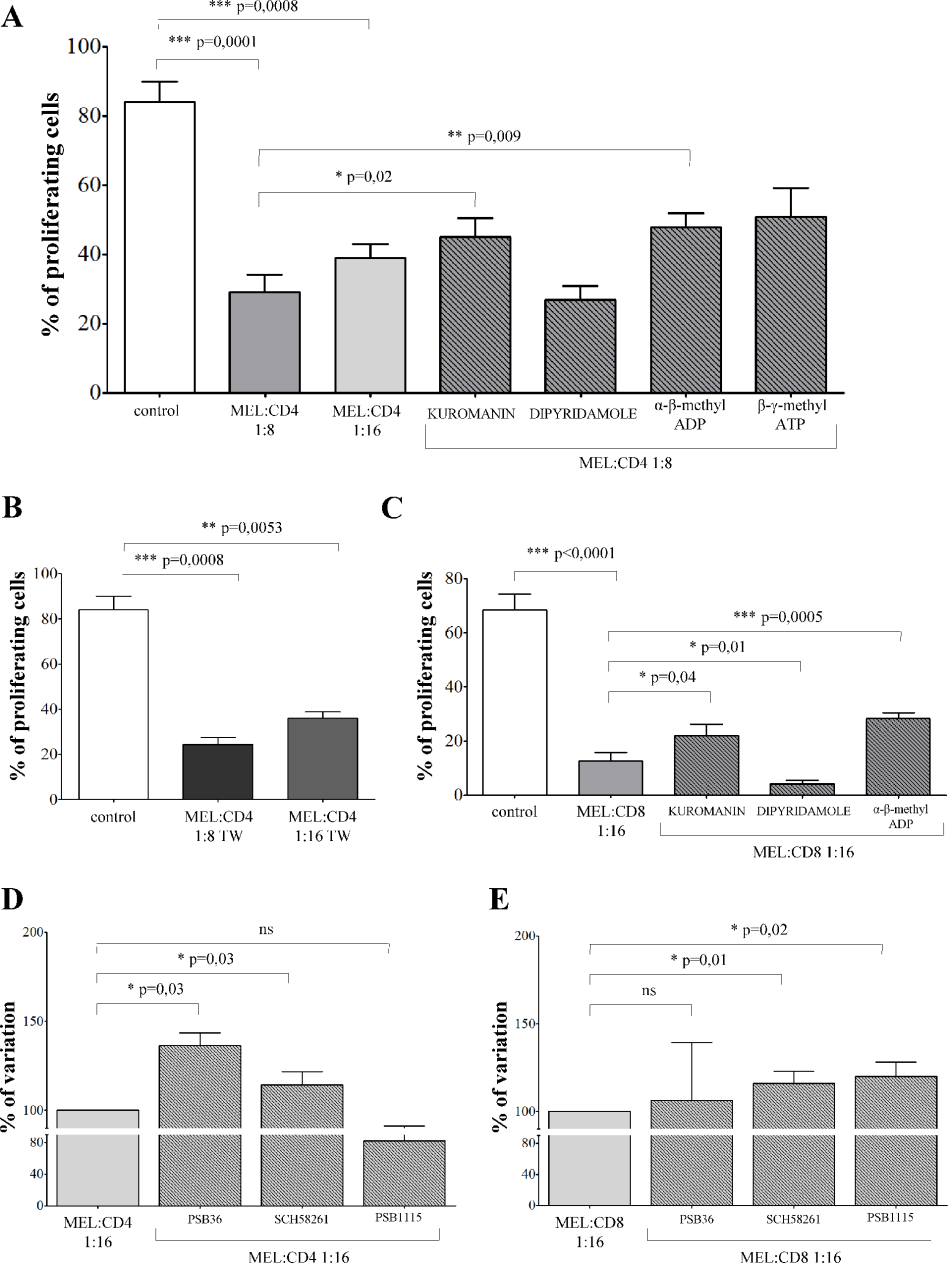
Inhibition of T cell proliferation by melanoma cell lines **Panel A.** CD4^+^ T cells stimulated with anti-CD3/CD28 beads (white bar) were co-cultured with melanoma cell lines at melanoma:T cell ratios of 1:8 (grey bar), 1:16 (light grey bar) or 1:8 in the presence of inhibitors (stripped bars). T cell proliferation was assessed by CFSE dilution using a flow cytometer. Results are expressed as % of proliferating cells. Mean of 12 experiments ± SD is shown. *p* values are indicated where differences are statistically significant. **Panel B.** CD4^+^ T cells stimulated with anti-CD3/CD28 beads (white bar) were co-cultured with melanoma cell lines at melanoma:T cell ratios of 1:8 (grey bar) or 1:16 (light grey bar) using a transwell system. T cell proliferation was assessed by CFSE dilution using a flow cytometer. Results are expressed as % of proliferating cells. Mean of 6 experiments ± SD is shown. *p* values are indicated where differences are statistically significant. **Panel C.** CD8^+^ T cells stimulated with anti-CD3/CD28 beads (white bar) were co-cultured with melanoma cell lines at melanoma:T cell ratios of 1:16, in the presence (stripped bars) or absence (grey bar) of inhibitors. T cell proliferation was assessed by CFSE dilution using a flow cytometer. Results are expressed as % of proliferating cells. Mean of 8 experiments ± SD is shown. *p* values are indicated where differences are statistically significant.

Proliferation of CD4^+^ T cells (% of proliferating cells ± SD: 84.06 ± 20.27) was significantly inhibited in the presence of melanoma cells at 1:8 (29 ± 17.1, *p* = 0.0001) or 1:16 (38.9 ± 11, *p* = 0.0008) melanoma:CD4^+^ T cell ratios (Figure 3, panel A). T cell proliferation was significantly restored in the presence of kuromanin (45.06 ± 18.1, *p* = 0.02) and α-β-methyl ADP (47.8 ± 13.54, *p* = 0.009). These findings indicate that CD38 and CD73 play key roles in the inhibition of T cell proliferation.

In order to assess whether inhibition of CD4^+^ T cell proliferation is mediated by the release of soluble factors by melanoma cells, the same experiments were performed using a transwell system. CD4^+^ T lymphocytes proliferation (% of proliferating cells ± SD: 84.06 ± 20.27) was significantly inhibited in the presence of melanoma cells either at 1:8 (24.28 ± 7.75, *p* = 0.0008) or 1:16 (36.3 ± 6.89, *p* = 0.005) melanoma:CD4^+^ T cell ratios. We conclude that the inhibition observed was prevalently mediated by soluble factors (Figure 3, panel B).

Similar experiments were performed using CD8^+^ T lymphocytes. CD8^+^ T cell proliferation (% of proliferating cells ± SD: 68.35 ± 16.95) was significantly inhibited in the presence of melanoma cells at 1:16 melanoma:CD8^+^ T cells ratio (12.61 ± 8.9, *p* < 0.0001, Figure 3, panel C). The observed inhibition was partially reverted by pre-treating melanoma cells with kuromanin (22.05 ± 12.1, *p* = 0.04) and α-β-methyl ADP (28.36 ± 6.2, *p* = 0.0005). The roles of CD38 and CD73 were therefore confirmed on this population, too. In addition, pre-treatment of melanoma cells with dipyridamole significantly increased the inhibition of T cell proliferation (4.1 ± 3.7, *p* = 0.01). This inhibitor is reported to increase extracellular concentrations of ADO by inhibiting nucleoside transporter. Apparently, ADO is more effective on CD8^+^ than on CD4^+^ T cell populations.

Additional experiments were performed to demonstrate unambiguously that the inhibition of T cell proliferation induced by melanoma cells was mediated by ADO. To this end, T cells were pre-treated with specific antagonists of ADORA1 (PSB36), ADORA2a (SCH58261) and ADORA2b (PSB1115) before being cultured with melanoma cells. As shown in Figure 3, panel D, proliferation of CD4^+^ T cells was significantly restored in the presence of PSB36 (% of variation ± SD: 100 vs 136.4 ± 12.84, *p* = 0.03) and SCH58261 (100 vs 114.2 ± 13.03, *p* = 0.03), but not of PSB1115 (100 vs 82.23 ± 15.46). In contrast, proliferation of CD8^+^ T cells was significantly restored in the presence of SCH58261 (% of variation ± SD: 100 vs 116.1 ± 13.54, *p* = 0.01) and PSB1115 (100 vs 120.1 ± 16.43, *p* = 0.01), but not of PSB36 (100 vs 106.3 ± 65.45). Taken together, these data confirmed that the inhibition of proliferation was mediated by ADO, mainly through the interaction with ADORA1 and A2a on CD4^+^ T cells, and with ADORA2a and A2b in CD8^+^ T cells.

### Central and effector memory T cells are the main targets of the inhibitory effects of melanoma cells

The next step was to investigate whether melanoma cells might have different effects on the T cell subsets. Co-culture experiments using each purified T cell subset at 1:16 melanoma: T cell ratio were performed to answer this issue.

**Figure 4:**
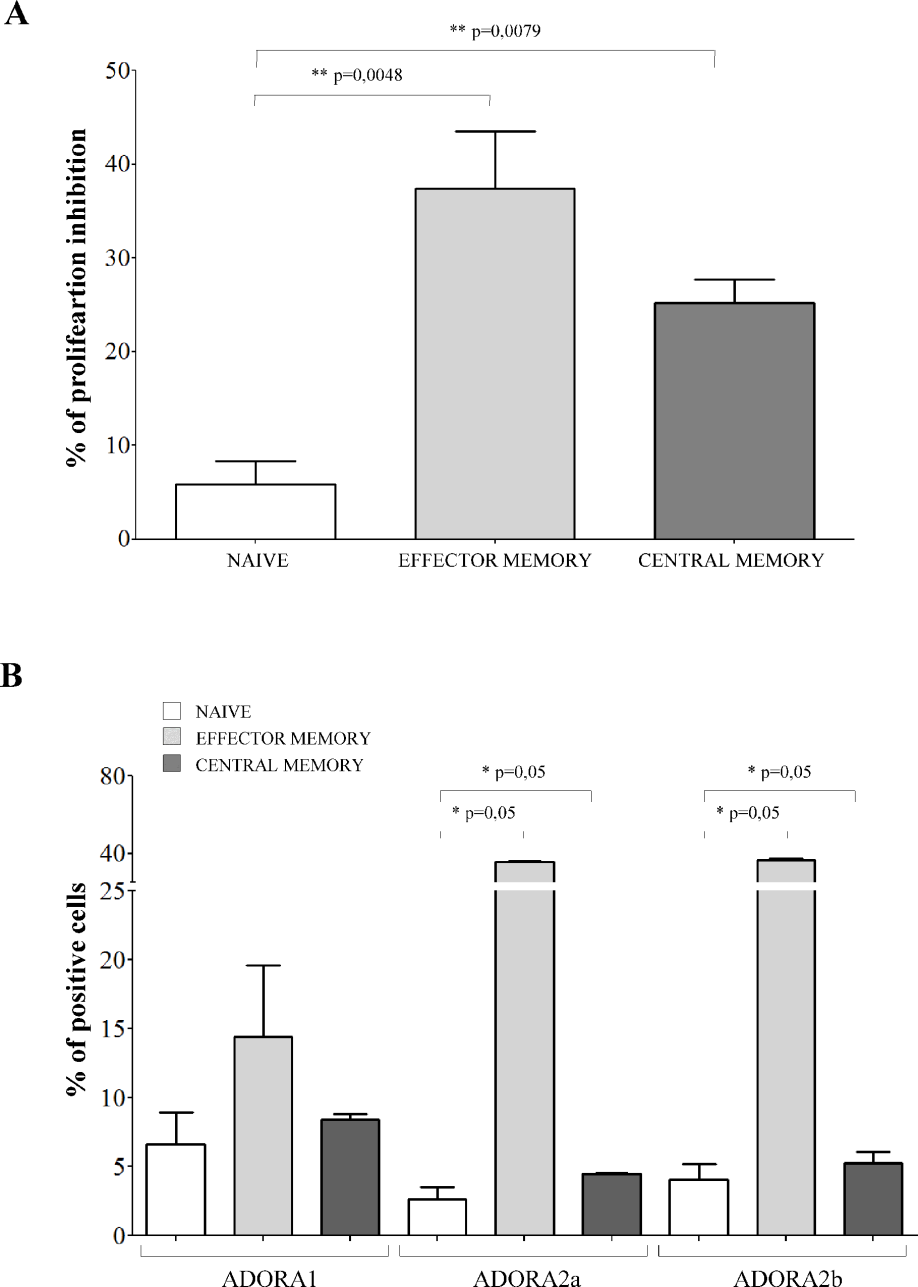
Inhibition of cell proliferation and expression of ADO receptors in CD4+ T cell subsets **Panel A.** Naïve (white bars), effector memory (light grey bars) and central memory (grey bars) CD4^+^ T lymphocytes were isolated and stimulated with anti-CD3/CD28 beads in the presence or absence of melanoma cell lines at 1:16 melanoma:T cell ratio. T cell proliferation was assessed by CFSE dilution using a flow cytometer. Results are expressed as % of inhibition, calculated as follows: *% of proliferating cells in the absence of melanoma cells - % of proliferating cells in the presence of melanoma cells/% of proliferating cells in the absence of melanoma cells*. Mean of 6 experiments ± SD is shown. *p* values are indicated where differences are statistically significant. **Panel B.** ADORA1, A2a, A2b was investigated by flow cytometry on purified CD4^+^ T cells, gating on naïve (white bars), effector memory (light gray bars) and central memory (grey bars). Data are expressed as % of positive cells. Mean of four different experiments ± SD is shown. *p* values are indicated where differences are statistically significant.

The inhibitory effects exerted by melanoma cells were tested on CD4^+^CD27^+^CD45RA^+^ naïve T cells, CD4^+^CD27^+^CD45RA^−^ central memory T cells and CD4^+^CD27^−^CD45RA^−^ effector memory T cells. Terminally differentiated effector CD4^+^CD27^−^CD45RA^+^ T cells were not included because of their limited presence in the peripheral blood of normal individuals (range 1–5%). Inhibition of CD4^+^ T cell proliferation in the presence of melanoma cell lines resulted significantly lower in naïve cells (% of inhibition ± SD: 5.82 ± 5.04) than in effector memory (37.42 ± 14.86, *p* = 0.0048) and central memory (25.17 ± 5.7, *p* = 0.0079) CD4^+^ T cells (Figure 4, panel A).

Next, we asked whether these differential effects might be related to a different expression of ADORs in these different subsets. ADORA1 was expressed similarly in the three different subsets (Figure 4, panel B). In contrast, ADORA2a and ADORA2b display a peculiar pattern of expression: higher in naïve cells (% positive cells ± SD: ADORA2a 32.16 ± 15.51; ADORA2b 71.69 ± 7.04) but lower in effector memory (ADORA2a 10.59 ± 8.06, *p* = 0.05; ADORA2b 32.94 ± 22.75, *p* = 0.05) and central memory (ADORA2a 2.61 ± 1.57, *p* = 0.05; ADORA2b 11.23 ± 2.18, *p* = 0.05) CD4^+^ T cells (Figure 4, panel B).

**Figure 5:**
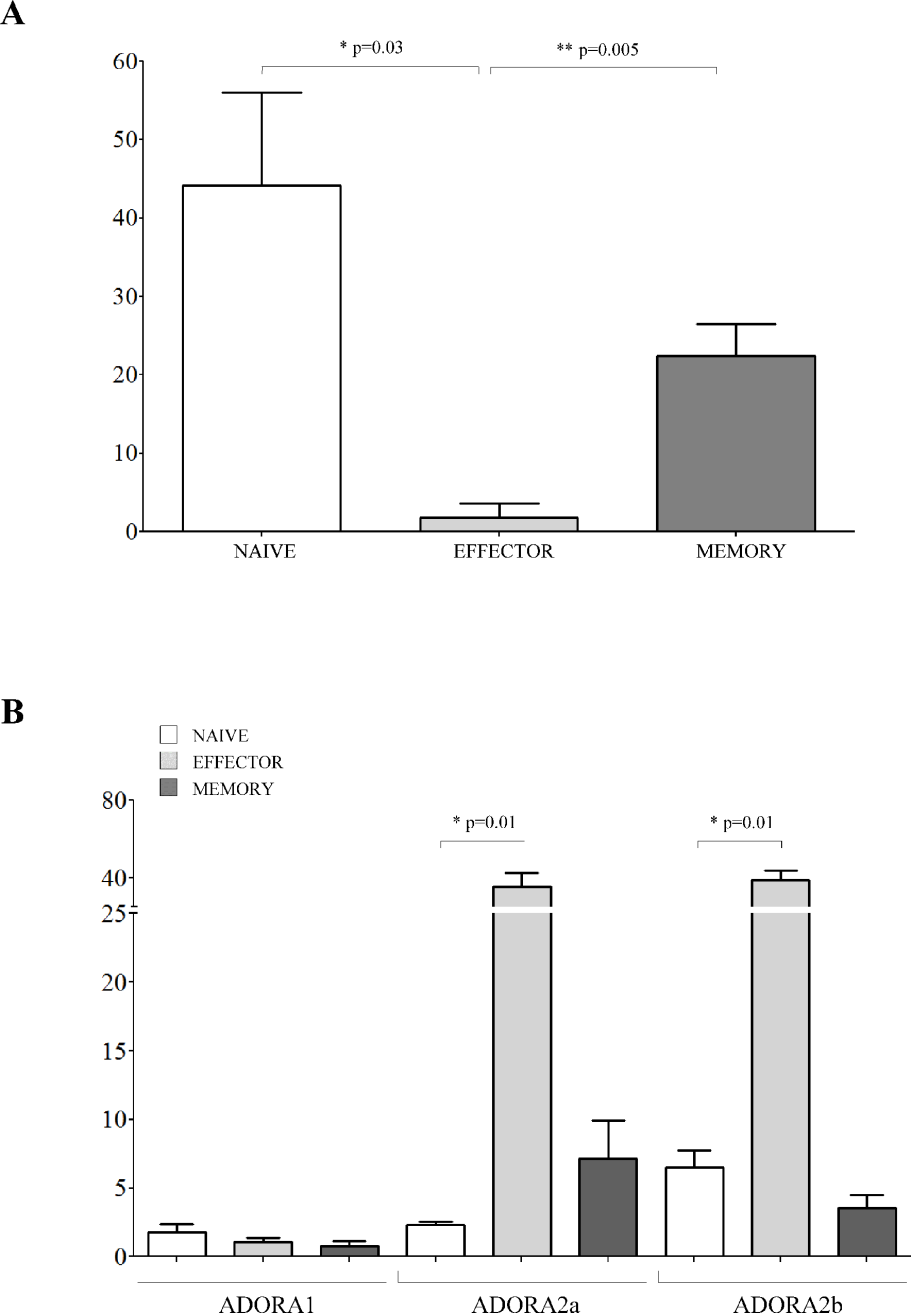
Inhibition of cell proliferation and expression of ADO receptors in CD8^+^ T cell subsets **Panel A.** Naïve (white bars), effector (light grey bars) and memory (grey bars) CD8^+^ T lymphocytes were isolated and stimulated with anti-CD3/CD28 beads in the presence or absence of melanoma cell lines at 1:16 melanoma:T cell ratio. T cell proliferation was assessed by CFSE dilution using a flow cytometer. Results are expressed as % of inhibition, calculated as follows: *% of proliferating cells in the absence of melanoma cells - % of proliferating cells in the presence of melanoma cells/% of proliferating cells in the absence of melanoma cells*. Mean of 6 experiments ± SD is shown. *p* values are indicated where differences are statistically significant. **Panel B.** ADORA1, A2a, A2b was investigated by flow cytometry on purified CD8^+^ T cells, gating on naïve (white bars), effector (light gray bars) and memory (grey bars). Data are expressed as % of positive cells. Mean of four different experiments ± SD is shown. *p* values are indicated where differences are statistically significant.

The effects of co-colture with melanoma cells was next evaluated on CD8^+^CD27^+^CD45RA^+^ naïve T cells, CD8^+^CD27^+^CD45RA^−^ memory T cells and CD8^+^CD27^−^CD45RA^−^ effector T cells. The percent inhibition of CD8^+^ T cell proliferation in the presence of melanoma cell lines was significantly lower in effector cells (% of inhibition ± SD: 1.83 ± 3.17) than in memory (22.38 ± 4.15, *p* = 0.005) and naive (44.12 ± 11.9, *p* = 0.03) CD8^+^ T cells (Figure 5, panel A). The expression of ADORs was also analyzed on the different CD8^+^ T cell subsets. CD8^+^ T cell subsets expressed similarly low levels of ADORA1. In contrast, ADORA2a and ADORA2b expression was higher in effector cells (% of positive cells ± SD: ADORA2a 35.54 ± 7.27; ADORA2b 39.13 ± 4.74) than in memory (ADORA2a 7.16 ± 2.78, *p* = 0.01; ADORA2b 3.58 ± 0.94, *p* = 0.01) and naive (ADORA2a 2.35 ± 0.23, *p* = 0.01; ADORA2b 6.52 ± 1.25, *p* = 0.01) CD8^+^ T cells (Figure 5, panel B).

Overall, these findings support the view that the melanoma cells are characterized by a selective ability to inhibit different subsets of CD4^+^ and CD8^+^ T cells. Furthermore, each subset is characterized by a particular pattern of expression of ADORA2a and A2b.

### Effects of ADO-mediated intracellular signaling in naïve and memory CD4^+^ T lymphocytes

**Figure 6:**
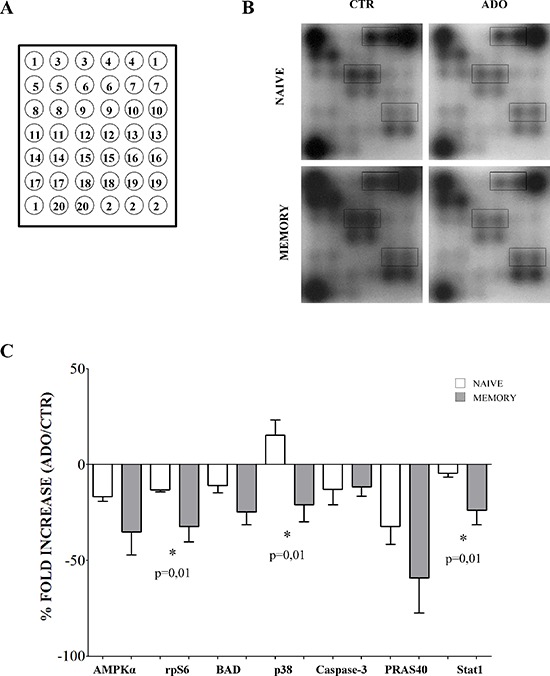
Evaluation of intracellular signaling in naïve and memory CD4^+^ T cells Intracellular signaling has been evaluated in naïve and memory CD4^+^ T cells stimulated with anti-CD3/CD28 beads in the presence (ADO) or absence (CTR) of ADO, using an intracellular signaling array kit. Target map is shown in **panel A.** and include the following proteins: 1) positive control, 2) negative control, 3) ERK1/2, 4) Stat1, 5) Stat3, 6) and 7) Akt, 8) AMPKα, 9) S6 ribosomal protein, 10) mTOR, 11) HSP27, 12) BAD, 13) p70 S6 kinase, 14) PRAS40, 15) p53, 16) p38, 17) SAPK/JNK, 18) PARP, 19) caspase-3, 20) GSK-3β. A representative experiment is shown in **panel B.** Proteins whose modulation was significantly different between naïve and memory T cells (Stat1, S6 ribosomal protein and p38) are highlighted. **Panel C.** shows modulation of phosphorylation of different factors in the presence of ADO in naïve (white bars) and memory (grey bars) CD4^+^ T cells. Results are expressed as % of fold increase, calculated as follows: ((relative intensity of spot obtained in the presence of ADO - relative intensity of spot obtained in the absence of ADO)/relative intensity of spot obtained in the absence of ADO)) *100. Mean ± SD of four independent experiments is shown.

We hypothesize that the inhibition of proliferation induced by ADO in naïve and memory T cells might reflect distinct ADO-mediated modulation of intracellular signaling in the two cell populations. We therefore investigated the phosphorylation of multiple key factors involved in cell signaling by treating naïve and memory CD4^+^ T cells with anti-CD3/anti-CD28 beads, in the presence (or absence) of ADO, using a specific antibody array. A scheme of this array is reported in Figure 6, panel A, while the list of proteins analyzed is reported in the corresponding figure legend.

Figure 6, panel B (representative experiment) and C (mean of 4 independent experiments) shows that ADO inhibits the phosphorylation of AMPK-α (% fold increase, mean ± SD: naïve −16.74 ± 2.44, memory −35.11 ± 12.02), S6 ribosomal protein (naïve −13.08 ± 1.14, memory −32.21 ± 8.02), BAD (naïve −10.96 ± 3.68, memory −24.74 ± 6.54), caspase-3 (naïve −12.89 ± 8.04, memory −11.61 ± 4.71), PRAS40 (naïve −32.29 ± 9.3, memory −59.1 ± 18.23) and STAT1 (naïve −4.38 ± 2.07, memory −23.76 ± 7.5). Conversely, p38 phosphorylation was increased in naïve (% of fold increase, mean ± SD: 15.43 ± 7.87) and inhibited in memory (−20.87 ± 8.8) CD4^+^ T cells (Figure 6, panel B and C).

The phosphorylation profiles of S6 ribosomal protein, p38 and Stat1 were significantly different in naïve and memory CD4^+^ T cells (highlighted in Figure 6, panel B, *p* = 0.01). Memory T cells displayed lower phosphorylation levels of these factors; this may be consistent with the stronger inhibition of proliferation induced by ADO in this subset, as compared to naïve counterparts.

**Figure 7:**
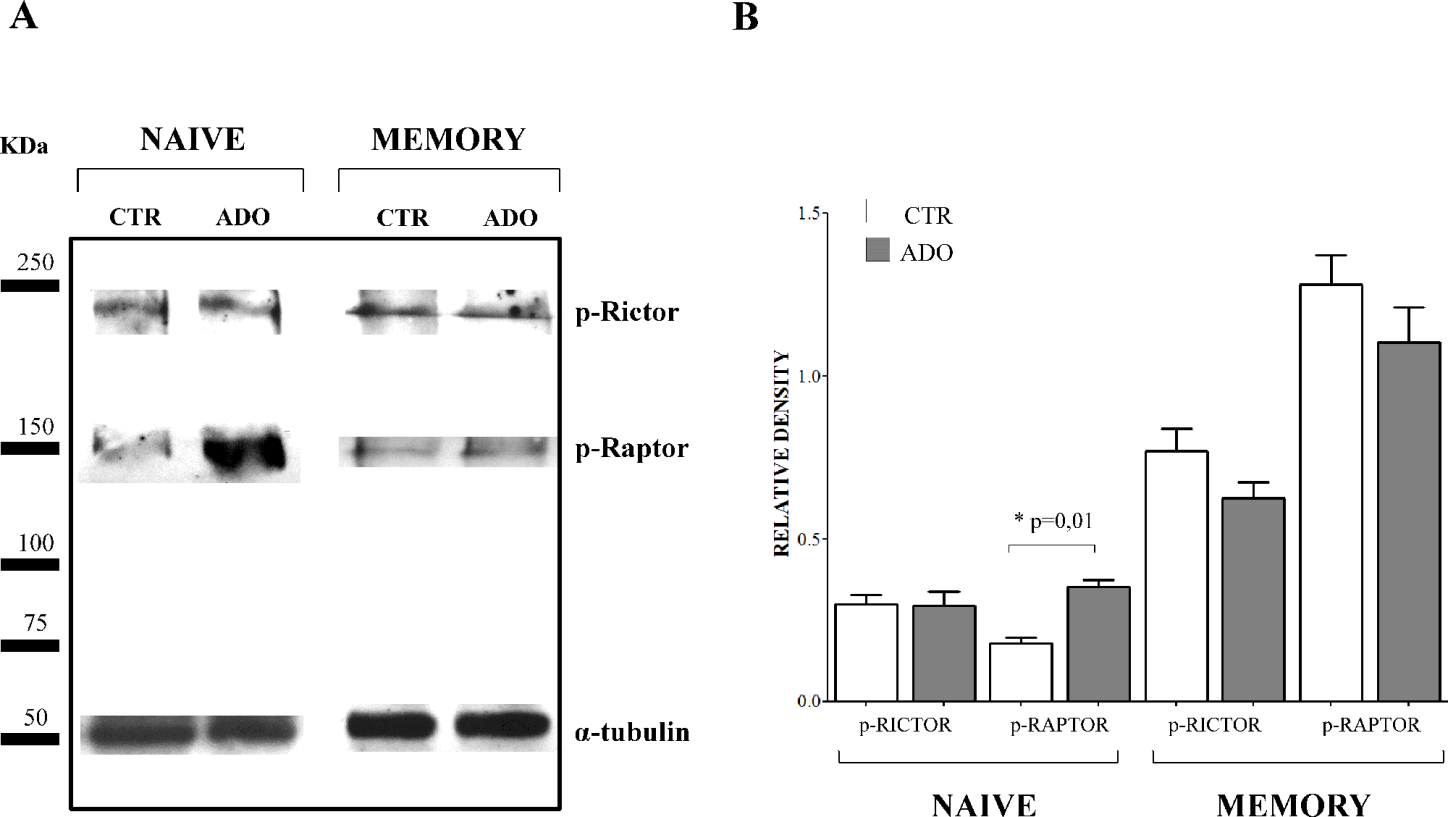
Evaluation of mTOR associated proteins in naïve and memory CD4^+^ T cells Phosphorylation of Rictor and Raptor has been evaluated by western blot analysis in naïve and memory CD4^+^ T cells stimulated with anti-CD3/CD28 beads in the presence (ADO, grey bars) or absence (CTR, white bars) of ADO. A representative experiment is shown in **panel A.** Mean of four different experiments ± SD is shown in **Panel B.** Results are expressed as relative density, calculated as follows: *(density of the specific protein/density of housekeeping protein)*.

Since mTOR pathway is essential for T lymphocyte proliferation, we further investigated the phosphorylation of regulatory associated protein of mTOR (Raptor) and rapamycin-insensitive companion of mTOR (Rictor) on the same protein extracts from naïve and memory CD4^+^ T cells. As shown in Figure 7, panel A (a representative experiment) and B (mean of four different experiments), the phosphorylation of Rictor was unaffected by ADO treatment, either in naïve (relative density: ctr 0.3 ± 0.05, ADO 0.29 ± 0.08) or memory (ctr 0.77 ± 0.13, ADO 0.62 ± 0.09) CD4^+^ T cells. Conversely, phosphorylation of Raptor was significantly increased by ADO treatment in naïve (ctr 0.18 ± 0.03, ADO 0.35 ± 0.04, *p* = 0.01) but not in memory (ctr 1.28 ± 0.18, ADO 1.10 ± 0.21) CD4^+^ T cells. These data further support the concept that intracellular signalling is differentially modulated by ADO in naïve and memory T cells.

## DISCUSSION

Several independent studies indicate that the production of ADO is a feature acquired by different solid tumors, and that the small or virtual spaces at the interface between the tumor and host organs, referred to as the microenvironment, are characterized by high levels of ADO [4, 5, 16, 24, 33, 34]. These attributes are now considered to make up a refined strategy on the part of tumor cells to exploit the ADO and its uptake by immune effector cells to circumvent immune defences [5, 6]. ADO can be produced from ATP or alternatively from NAD^+^ which are metabolized by a panel of ectoenzymes present on the surface of tumor cells and expressed by local or infiltrating effector lymphocytes [25, 33, 35].

Separate groups have demonstrated that CD73 promotes tumor growth in experimental tumor models [6, 18], including melanoma. However, CD73 knock-out mice are resistant to tumor metastasis and display a potent anti-tumor immune response [18]. The *CD73* gene is methylated in normal melanocytes and primary melanoma lesions, but it is epigenetically up-regulated during melanoma progression. This further support a role of this molecule during tumorigenesis [13].

Given the functions of ADO in the immune system, the set of the different receptors specific for ADO is crucial both for the growth of tumor and for tumor-mediated immunosuppression; indeed, metastases are limited when these receptors are blocked [25] or knocked-out [24]. Whether melanoma cells can directly release ADO and whether ADO can inhibit the functions of T lymphocytes is still unclear.

This study demonstrates that primary melanoma cells are equipped with both the canonical (CD39/CD73) and the alternative [2, 3] (CD38/CD157/CD203a(PC-1)/CD73) ectoenzymatic pathways for producing ADO at the interface between tumor and host. All these ectoenzymes are functional in the primary cell lines of human melanoma analyzed. This finding shows that these cells may use ATP and NAD^+^ to produce ADO. CD73 operates as a sort of checkpoint. As a matter of fact, the maximum release of ADO was obtained after adding AMP, which suggests that CD73 is the most active of the ectoenzymes. This is also corroborated by the finding that CD73 is the most expressed on all melanoma cells analyzed.

The variability in ADO production observed after providing different substrates is related to the heterogeneous expression of ectoenzymes by primary melanoma cells. Indeed, the maximum ADO production from ATP (a substrate for CD39 and CD203a/PC-1) and ADPR (a substrate for CD203a/PC-1) was achieved in CD39^high^CD203a/PC-1^high^ cell lines. Consistent with this observation, ADO production from AMP and NAD^+^ was comparable in all cell lines expressing similar levels of CD73 and CD38 (specific enzymes for AMP and NAD^+^ respectively).

The immunosuppression exerted by ADO on immune effector cells can be obtained in different ways [5, 6, 34]. For instance, ADO can interfere signals mediated by IL-2 receptor by dephosphorylating SHP-2 and STAT-5 [36]. The consequence is inhibition of TCR-triggered proliferation and CD25 up-regulation [37]. Other modalities of ADO functionalities related to the inhibition of cytotoxicity and cytokine production by specific anti-melanoma T cells have also been reported [8].

The original observations of this work are that: i) primary melanoma cell lines inhibit CD4^+^ and CD8^+^ T cell proliferation, ii) such inhibition is mediated by soluble factor(s), and iii) a key role is exerted by ADO. These conclusions are supported by substantial evidence. On the one hand, specific inhibitors of CD73 and CD38 partly (but significantly) restored T cell proliferation. Such proliferation was not fully restored in the presence of these inhibitors, probably because two alternative pathways for ADO production are operating on melanoma cells. However, we cannot exclude the contribution of other immunosuppressive mechanism(s). However, this indicates that the function of both molecules, cooperating in ADO production, is crucial for melanoma-driven inhibition of T cell proliferation.

Secondly, the key role of ADO in such effects on T cells was confirmed by the findings that i) CD8^+^ T lymphocytes proliferation was further dampened by dipyridamole, a specific inhibitor of nucleoside transporter, that causes ADO to accumulate in the extracellular milieu and ii) proliferation of T cells was significantly restored by pre-treating these cells with specific antagonists of ADORs. A third observation is that the anti-proliferative effects featured by melanoma cells was different on CD4^+^ and CD8^+^ T cell subsets. Consequently, proliferation was significantly impaired in CD4^+^ T cells, in effector and central memory, and – to a lesser extent −, in naïve T cells. Different results were obtained in CD8^+^ T cells, where proliferation of naïve and memory was inhibited more than in effector T cells. We hypothesize that this apparent discrepancy could be due to a selective expression of ADO receptors on these lymphocyte populations.

There are four ADOR subtypes (A1, A2a, A2b and A3), that belong to the G-protein-coupled seven-transmembrane family of cell-surface receptors [5]. A1 and A2a ADOR (Kd for ADO, ∼10^−8^ to 10^−7^ M) exhibit higher relative affinities for ADO than A2b and A3 ADOR (Kd for ADO, ∼10^−6^ to 10^−5^ M). ADOR subtypes are coupled to different combinations of G-protein family members, namely A1 ADOR to G_i_/G_o_, A2a ADOR to G_s_/G_olf_, A2b ADOR to G_s_/G_q_, and A3 ADOR to G_i_/G_q_. Accordingly, engagement of ADORA2a and A2b activates adenyl cyclase, leading to elevated levels of cellular cyclic AMP (cAMP). Instead, A1 and A3 stimulation inhibits adenyl cyclase, resulting in decreased cellular levels of cAMP. In addition, all ADOR subtypes couple to mitogen-activated protein kinase (MAPK) pathways. The final effects of extracellular ADO on lymphocyte function are dictated by the expression of distinct surface ADOR, as well as by their relative affinities for ADO.

The ADORA2a and A2b were differentially expressed by the subsets whose proliferation is less inhibited by melanoma cell lines (*i.e*. naïve CD4^+^ T cells and effector CD8^+^ T cells) when compared to other cell subsets. These receptors are endowed with anti-proliferative effects on T cells [38] by modulating cAMP, MAPK, ERK1-2 and JAK/STAT pathways. Thus, the correlation between high expression of these receptors and the low inhibition of proliferation induced by melanoma cells might be related to a different intracellular pathway activated by ADO in naïve CD4^+^ T cells and effector CD8^+^ T cells compared to the other subsets. Further evidence comes from the observation that the phosphorylation of S6 ribosomal protein, p38 and Stat1 in CD4^+^ T cells activated in the presence of ADO was significantly lower in memory than in naïve CD4^+^ T lymphocytes. S6 ribosomal protein is reported as a regulator of mRNA and cell proliferation [39], while p38 and Stat1 are key factors in signal transduction in response to cytokines and other stimuli [40, 41]. Thus, it is reasonable to conclude that the inhibition of memory CD4^+^ T lymphocytes proliferation mediated by ADO occurs through a different modulation of cell cytoplasmic signals. ADORA1 subtype, that is associated to a reduction in cellular cAMP levels, was similarly expressed by all T cell subsets.

**Figure 8:**
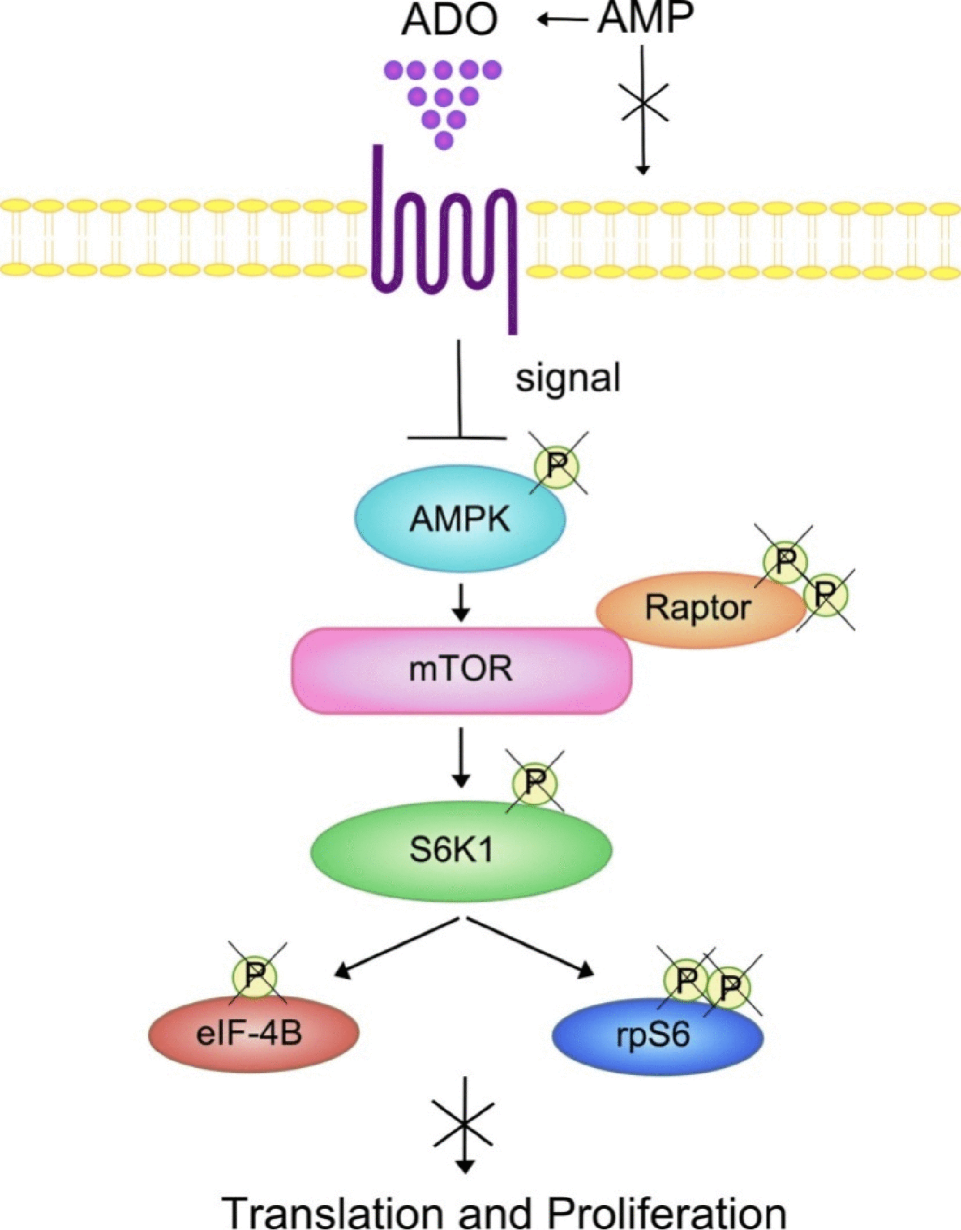
Summary of the effects of ADO on the AMPK/mTOR/p70S6K/rpS6 protein axis in T lymphocytes Positive signaling through mTOR, a downstream effector of AMPK, regulates protein translation and cell proliferation. mTOR activity can be monitored by the phosphorylation status of AMPK, S6K or its downstream substrate rp6S. ADO inhibition of the phosphorylation status of the AMPK/mTOR/S6K/rpS6 components of the signaling cascade may decrease lymphocyte protein synthesis and cell proliferation. *Abbreviations: AMPK, AMP-activated protein kinase; S6K, p70 ribosomal S6 kinase 1; elF4B, eukaryotic translation initiation factor; mTOR, mammalian target of rapamycin; Raptor, regulatory-associated protein of mTOR*.

Emerging evidence indicates that metabolic signals regulate AMP-activated protein kinase (AMPK) and mTOR thus directing T lymphocyte-mediated immune responses [37]. In line with this, AMPK activation regulates cell proliferation by inhibition of mTOR signalling [42]. AMPK is activated after phosphorylation of the α-catalytic subunit by the glycogen synthase kinase-3 (GSK3) induced by AMP, that in turn inhibits mTOR activity, a major regulator of translation initiation and cell proliferation. Thus, the conversion of AMP into ADO provokes antagonistic effects on AMPK activation. Some of the most characterized downstream effectors of mTOR is the 70 kDa ribosomal protein S6 kinase 1 (p70S6K) and S6rp. Non-phosphorylated AMPK might be unable to induce phosphorylation of essential components of mTOR pathway (S6rp, p70S6K) [43], with a reduction of cell proliferation (Figure 8). Phosphorylation of the AMPK/mTOR/p70S6K/rpS6 protein axis was also significantly lower in memory than in naïve CD4^+^ T cells, with reduced proliferation in the presence of melanoma cells. It is also known that AMPK-deficient CD8^+^ T cells produce more inflammatory cytokines than wild-type T cells. This feature is not present in AMPK-deficient CD4^+^ T cells, suggesting that AMPK is mainly a negative regulator of CD4^+^ T cell activation. Finally, we have demonstrated that the phosphorylation of Raptor was increased by ADO in naïve (but not in memory) T cells. Since this regulatory protein is involved in mTOR-mediated phosphorylation of 4E-BP1 and p70 S6K, leading to cell cycle progression, these data may further explain the very low inhibition of T cell proliferation induced by ADO in naïve CD4^+^ T cells.

The results of this study demonstrate that primary melanoma cells inhibit T cell proliferation by producing ADO from distinct and functional ectoenzymatic pathways. Such inhibition is more effective on T cell subsets prevalently involved in anti-tumor immune response (i.e., central and effector memory CD4^+^ T cells and memory CD8^+^ T cells). The clinical relevance of this observation is that ADO is a key component of a strategy adopted by human melanoma cells to escape the specific immune response. These data may pave the way to the development of novel therapeutic strategies for blocking ectoenzymes involved in ADO production, thereby restoring a local potent anti-tumor immune response.

## MATERIALS AND METHODS

### Melanoma cell lines and T cell isolation

MECA, METRAV, MEPA, MECO, MEMO and MEOL are primary melanoma cell lines established from patients who underwent surgical resection of skin or lymph node metastases at Istituto Nazionale Ricerca sul Cancro (Genoa), as previously described [28]. The FO1 melanoma cell line was purchased by ATCC. Melanoma cell lines were cultured in RPMI medium (Sigma Chemical Company) supplemented with 10% FBS (Sigma).

PB samples were obtained from normal donors afferent at Blood Bank of Istituto Giannina Gaslini (Genoa). CD4^+^ and CD8^+^ T cells were isolated by incubating whole blood samples with RosetteSep^TM^ Human CD4^+^ T cell or CD8^+^ T cell enrichment cocktail (StemCell Technologies) following manufacturer's protocol. Untouched CD4^+^ T cell or CD8^+^ T cells were then obtained by Ficoll-Hypaque (Sigma) density gradient centrifugation.

Naïve, effector memory and central memory CD4^+^ T cell subsets and naïve, effector and memory CD8^+^ T cell subsets were isolated from PB mononuclear cells (MNC) obtained by Ficoll-Hypaque (Sigma Chemical Company) density gradient centrifugation, using the following isolation kits (Myltenyi Biotec): Pan naïve T cells Isolation kit, naive CD4^+^ T Cell Isolation Kit II, CD4^+^ Effector Memory T Cell Isolation Kit, CD8^+^CD45RA^+^ Effector T Cell Isolation Kit, CD4^+^ Central Memory T Cell Isolation Kit and CD8^+^ Memory T Cell Isolation Kit. Cells were isolated following manufacturer's protocol. Naïve CD8^+^ T cells were obtained using pan naïve T cells Isolation kit and subsequently naive CD4^+^ T Cell Isolation Kit II, collecting the negative fraction.

For intracellular signaling experiments, naïve CD4^+^ T cells were obtained as described above from untouched total CD4^+^ T cells. Memory CD4^+^ T cells were obtained as positive fraction from the same cell preparation.

### Flow cytometric analysis

Evaluation of the expression of ectoenzymes was performed on melanoma cell lines using the following monoclonal (m)Abs generated in our lab and FITC- or APC-conjugated by Aczon (Bologna, Italy): anti-CD38 (#IB4), anti-CD73 (#CB73), anti-CD157 (#SY/11B5), anti-CD203a/PC-1 (#3E8, kindly provided by J. Goding). CD39 expression was analyzed using anti-CD39 PE-Cy7 mAb (eBioscience). FITC- or APC-conjugated irrelevant isotype-matched mAbs were purchased from Beckman Coulter.

The expression of ADOR was evaluated on total CD4^+^ or CD8^+^ T cells isolated as previously described from five normal donors, gating on naïve (CD27^+^CD45RA^+^), CD4^+^ central memory/CD8^+^ memory (CD27^+^CD45RA^−^) or CD4^+^ effector memory/CD8^+^ effector (CD27^−^CD45RA^−^) T cells, using the following antibodies: anti-CD45RA PC7, anti-CD27 APC (eBiosciences), purified rabbit polyclonal anti-ADORA1 (LifeSpan Biosciences, Inc.), rabbit polyclonal anti-ADORA2a (Thermo Scientific), goat polyclonal anti-ADORA2b (Santa Cruz Biotechnology). FITC-conjugated goat-anti rabbit Ig (Abcam) and swine-anti goat Ig (Life Technologies) were used as secondary reagents.

Cells were run on Gallios cytometer and analyzed using Kaluza software (Beckman Coulter). Data were expressed as mean relative of fluorescence intensity (MRFI), obtained as follows: *mean fluorescence obtained with specific mAb/mean fluorescence obtained with irrelevant isotype-matched mAb*. In some experiments, data were expressed as percentage of positive cells.

### ADO production by melanoma cell lines

Melanoma cell lines (5×10^5^ cells/well) were cultured in 0,1% glucose PBS at 37°C and 5% CO_2_ in 24 well plates (Costar Corning), in the presence (or absence) of AMP, ADPR, ATP or NAD^+^ (20 or 50 μM). In some experiments, cells were treated for 30 min with EHNA [44] (inhibitor of ADO deaminase, 100 μM) before being cultured with the substrates. Supernatants were collected after 5, 30 or 45 min and acetonitrile (ACN) was immediately added (1:2 ratio at 4°C) to stabilize ADO. Samples were then centrifuged at 12,000 rpm and supernatants were collected and stored at −80°C until use. The presence of ADO, AMP, ATP and NAD^+^ was investigated by HPLC assay.

### HPLC analysis

Chromatographic analysis was performed with an HPLC System (Beckman Gold 126/166NM, Beckman Coulter) equipped with a reverse-phase column (Synergi Fusion C18, 5 μm; 150 × 4.5 mm, Phenomenex). The nucleotides and nucleosides were separated using a pH 5.1 mobile-phase buffer (0.125 M citric acid and 0.025 M KH_2_PO_4_) along with 8% acetonitrile (Sigma Aldrich) over 10 min at a flow rate of 0.8 mL/min. UV absorption spectra were measured at 254 nm. HPLC-grade standards used to calibrate the signals were dissolved in AIM V serum-free medium (Invitrogen, Paisley, UK), pH 7.4, 0.2 μm sterile-filtered and injected in a buffer volume of 20 μL. The retention times (R_t_, in min) of standards were: AMP, 2.15; NAD^+^, 2.8; ADPR, 3.2; and ADO; 5.56. Peak integration was performed using a Karat software (Beckman Coulter).

ACN-treated melanoma cell supernatants (see above) were evaporated by speed-vac, reconstituted in mobile-phase buffer, and assayed by HPLC.

The qualitative identity of HPLC peaks was confirmed by co-migration of known reference standards. The presence of ADO was also confirmed by spiking standard (50 μM ADO), followed by chromatography. Quantitative measurements were inferred by comparing the peak area of samples with calibration curves for peak areas of each standard compound. Product concentrations were expressed as nmol/30 min/number of cells (5×10^5^ cells).

### T cell proliferation

T cell proliferation was assessed by Carboxyfluorescein succinimidyl ester (CFSE) dilution assay. Briefly, T cells were stained with CFSE (Invitrogen, 1 μg/ml, 15 min at 37°C), washed, and then cultured in RPMI medium added along with 10% FBS at 37°C and 5% CO_2_, alone or in the presence of anti-CD3/anti-CD28 mAb coated beads (T cell activation/expansion kit, Miltenyi Biotec). Stimulated T cells were cultured in 24 or 96 flat-bottom well plates (Costar Corning) in the presence or absence of melanoma cell lines (at 1:8 or 1:16 melanoma:T cell ratios). In some experiments, melanoma cell lines were treated for 30 min with the following specific inhibitors before being cultured with T cells at 1:16 melanoma:T cell ratio: 10 μM kuromanin [45] (an inhibitor of CD38, Sigma Aldrich), 50 μM dipyridamole [46] (an inhibitor of nucleoside transporter, Sigma Aldrich), 300 μM α-β-meADP [47] (an inhibitor of CD73, Sigma Aldrich) and 300 μM β-γ-meATP [48] (an inhibitor of CD203a/PC-1, Sigma Aldrich). In some experiments, CD4^+^ T cells were cultured 24 well plates (Costar Corning) and melanoma cell lines were cultured in the upper chamber of a transwell system (Costar Corning), at 1:8 or 1:16 melanoma:CD4^+^ T cell ratios. In some experiments, CD4^+^ or CD8^+^ T cells were treated for 30 min with the following specific inhibitors (all purchased from Tocris Bioscience) before being cultured with melanoma cells at 1:16 melanoma:T cell ratio: SCH58261 (A2a Receptor Antagonist), PSB1115 (A2b Receptor Antagonist) and PSB36 (A1 Receptor Antagonist).

After 6 days, cells were harvested and washed, and then stained with PE-conjugated anti-CD4 or anti-CD8 mAbs (Beckman Coulter). After additional washes, cells were run on Gallios cytometer, and CFSE dilution was analyzed gating on CD4^+^ or CD8^+^ cells, using Kaluza software (Beckman Coulter). In some experiments, cells were run on MACSQuant Analyzer (Miltenyi Biotec) and analyzed using FlowJo X Data Analysis Software (FlowJo LLC). Data were expressed as % of proliferating cells. In some experiments, data were expressed as % of proliferation inhibition, calculated as follows: *% of proliferating cells in the absence of melanoma cells - % of proliferating cells in the presence of melanoma cells/% of proliferating cells in the absence of melanoma cells*. In some experiments, data were expressed as % of variation, calculated as follows: *(% of proliferating cells in the presence of inhibitor/% of proliferating cells in the absence of inhibitor) x 100*.

### Intracellular signaling

Naïve and memory CD4^+^ T cells were stimulated overnight with anti-CD3/anti-CD28 beads (Myltenyi Biotec) following manufacturer's protocol. Cells were then washed, resuspended in RMPI medium, and incubated at 37°C in the presence or absence of 50 μM ADO (for 15 min). IL-2 (Proleukin, Novartis Pharm S.p.A., 100 U/ml) was then added and cells were incubated at 37°C for additional 15 min. Cells were then washed with PBS and lysed using PathScan Intracellular Signaling Lysis Buffer (Cell Signaling Technologies). Protein concentration was assessed using a BCA assay (BioRad). Cell lysates were diluted at a protein concentration of 0.7 mg/ml and intracellular signaling was investigated using PathScan Intracellular Array kit (Cell Signaling Technlogies) following manufacturer's protocol. In some experiments, protein lysates (20 μg per lane) were resolved on SDS 10% polyacrylamide gels and were transferred to nitrocellulose membranes. The membranes were then incubated with anti-Phospho-Raptor or anti-Phospho-Rictor rabbit polyclonal antibodies (Cell Signaling, MA, USA). Peroxidase-conjugated goat anti-rabbit polyclonal antisera was used as secondary reagent (Santa Cruz Biotechnology). Immune complexes were visualized with the use of a Supersignal West Pico Chemiluminescent Substrate (Pierce, Rockford, IL, USA) according to the manufacturer's instructions, and were normalized to internal controls (a mouse monoclonal antibody against alpha-tubulin, Cell Signaling).

Protein levels were quantified by scanning densitometry of the autoradiography films using VersaDoc 3000 Gel Imaging System (BioRad, Hercules, CA, USA) and normalized over the housekeeping protein levels (relative density). Results are expressed as % of fold increase, calculated as follows: [(relative intensity of spot obtained in the presence of ADO - relative intensity of spot obtained in the absence of ADO)/relative intensity of spot obtained in the absence of ADO)] ×100. In some experiments, data were expressed as relative density.

### Statistical analysis

Statistical analysis was performed using Prism 5.03 software (GraphPad Software). Gaussian distribution of data was analyzed using Kolmogorov-Smirnov test. The Student *t* test or Mann-Whitney test was used to compare data, depending on data distribution. The significance range as follows: *p < 0.05 (significant), **p < 0.005, and ***p < < 0.0005.
